# Finite element models with automatic computed tomography bone segmentation for failure load computation

**DOI:** 10.1038/s41598-024-66934-w

**Published:** 2024-07-17

**Authors:** Emile Saillard, Marc Gardegaront, Aurélie Levillain, François Bermond, David Mitton, Jean-Baptiste Pialat, Cyrille Confavreux, Thomas Grenier, Hélène Follet

**Affiliations:** 1 INSERM, LYOS UMR 1033, Université Claude Bernard Lyon 1, 69008 Lyon, France; 2 INSA-Lyon, CREATIS UMR5220, Université Claude Bernard Lyon 1, Villeurbanne, France; 3 Univ Eiffel, LBMC UMRT9406, Université Claude Bernard Lyon 1, 69622 Lyon, France; 4 Hospices Civils de Lyon, Lyon, France

**Keywords:** Risk factors, Bone cancer, Metastasis, Bone metastases, Computational models, Image processing, Machine learning

## Abstract

Bone segmentation is an important step to perform biomechanical failure load simulations on in-vivo CT data of patients with bone metastasis, as it is a mandatory operation to obtain meshes needed for numerical simulations. Segmentation can be a tedious and time consuming task when done manually, and expert segmentations are subject to intra- and inter-operator variability. Deep learning methods are increasingly employed to automatically carry out image segmentation tasks. These networks usually need to be trained on a large image dataset along with the manual segmentations to maximize generalization to new images, but it is not always possible to have access to a multitude of CT-scans with the associated ground truth. It then becomes necessary to use training techniques to make the best use of the limited available data. In this paper, we propose a dedicated pipeline of preprocessing, deep learning based segmentation method and post-processing for in-vivo human femurs and vertebrae segmentation from CT-scans volumes. We experimented with three U-Net architectures and showed that out-of-the-box models enable automatic and high-quality volume segmentation if carefully trained. We compared the failure load simulation results obtained on femurs and vertebrae using either automatic or manual segmentations and studied the sensitivity of the simulations on small variations of the automatic segmentation. The failure loads obtained using automatic segmentations were comparable to those obtained using manual expert segmentations for all the femurs and vertebrae tested, demonstrating the effectiveness of the automated segmentation approach for failure load simulations.

## Introduction

Bone metastasis can frequently be found on cancer patients, particularly those with primary breast or prostate cancer^1^, and are the cause of a variety of complications, among which pathological fractures can lead to a substantial decrease in the quality of life of the patients. Computed tomography (CT) is the imaging modality of choice for clinicians to evaluate the risk of pathological fractures, and although clinical scores, like Mirels score and SINS exist to evaluate fracture risk or bone instability in the case of femoral^2^ and vertebral^3^ metastasis respectively. Those scores either lack specificity^4–6^ or show limitations when the score is intermediate^7,8^.

To help the clinicians better evaluate the medical response, simulations based on finite element models are adequate to study the mechanical behavior of bones using CT-scans^9^. Failure load computation allows estimation of the fracture risk using information from the CT-scans along with mechanical properties. Several finite element models exist to perform failure load simulations on femurs^10–17^ and vertebrae^18–20^, but few on metastatic bones. The quality and precision of bone segmentations are essential to obtain a reliable failure load estimation of the bones under constraint. Having experts manually annotate data is usually a satisfactory way of obtaining high-quality segmentations, but the process, in addition to being dependent on operator reliability, is very tedious and time-consuming. When trying to simulate mechanical loads on the bones, the operator variability on segmentations can impact the results^21^ and hinder the reproducibility of the failure load computation. To obtain reproducible experiments and accurate simulations, automating the segmentation task is an important step. Automatic segmentation methods for femurs^17,22–26^, and vertebrae^27–30^ allow us to work towards automating failure load simulations on those bones. Convolutional neural networks are robust and allow to efficiently carry out segmentation tasks in the medical field^31,32^, showing great reliability and segmentation performance, but heavily depend on the size, annotation precision and image quality in the training dataset.

The objective of our study is to propose automatic segmentation methods for femurs and vertebrae, then evaluate the quality of the automatic segmentations not only in terms of images metrics using DICE score and Hausdorff distance, but also in terms of failure load results compared to expert manual segmentations, when used in our simulation pipeline. We investigated the effect of variations of the initial automatic segmentation on the numerical simulations, for femurs and vertebrae, to better study the sensibility of the finite elements models to the input segmentation.

## Results

### Automatic segmentations

As shown in Table 1, the segmentation scores obtained with our models indicate a good segmentation quality.

**Table 1:** Segmentation results on the primary femur dataset.

Task/dataset	Algorithm	DSC	HD (mm)
Femur segmentation MEKANOS database	U-Net 2D multi axial*	0.74 ± 0.09	49.86 ± 12.57
	U-Net 2D multi axial	0.93 ± 0.01	2.30 ± 0.82
	U-Net 3D	0.96 ± 0.01	2.20 ± 0.71
	nnUNet 3D fullres	0.96 ± 0.01	2.40 ± 0.84
Vertebrae segmentation VerSe 2019 & 2020	nnUNet 3D fullres—binary	0.95 ± 0.02	19.74 ± 36.54
	nnUNet 3D fullres—multiclass	0.89 ± 0.14	33.89 ± 58.09

*Without pre-processing.

The results highlight the importance of dedicated pre-processing when using custom U-Net architectures. For this task, the 3D models (nnUNet, custom 3D U-Net) outperform the 2D models, even when using a multi-axial approach. 3D U-Net and nnUNet perform very similarly, with only a slight improvement of HD for 3D U-Net.

Table 2 contains results of the nnUNet model on the secondary dataset with 16 femurs, with global and local metrics. The metrics obtained are close to the ones obtained previously, with a slightly higher DICE but a higher Hausdorff distance. The shaft is as expected the region segmented more accurately, but the model still achieves quality segmentation of the proximal part of the femur.

**Table 2:**
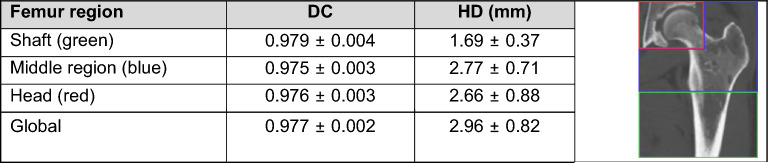
Segmentation results on our secondary femur dataset depending on the area.

For vertebrae segmentation, nnUNet proved very efficient, but the HD is much higher, due to small mislabeling of some vertebrae. The proximal part of the femur is more prone to segmentation errors than the distal part, but inaccuracies remain minimal. On vertebrae, the vertebral body and pedicle are less likely to be mislabeled or mis-segmented than the spinous or transverse process, where the delimitation between two vertebrae is more difficult to assess. The metrics obtained per vertebra are indicated in Supplementary Table S1. Those results show a disparity in the quality of the automatic segmentation depending on the vertebra, with transitional vertebra L6 and thoracic vertebrae T7 to T10 being more frequently mislabeled, causing a decrease of the DICE score for those vertebrae.

**Figure 1:**
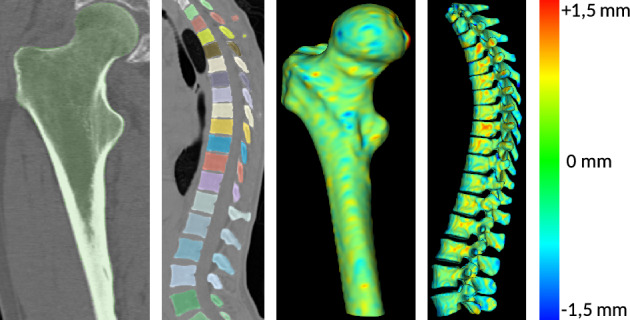
Automatic segmentations obtained with nnUNet (left) and pixels distance to the ground-truth (right).

Figure 1 shows automatic segmentations obtained with our approach for both femurs and vertebrae, as well as the differences obtained in terms of distance when comparing to expert manual segmentation.

### Failure load simulations

**Figure 2:**
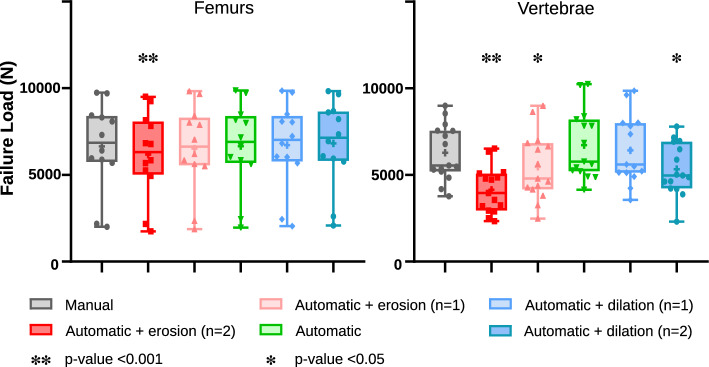
Simulated failure load depending on segmentation.

As shown in Table 3 and Fig. 2, the failure loads obtained for femurs, healthy or metastatic, using automatic segmentations are very close to those obtained with expert annotation.

**Table 3:** Comparison of image metrics and simulation results with manual segmentation.

		Automatic segmentation + erosion (2 pixels)	Automatic segmentation + erosion (1 pixel)	Automatic segmentation	Automatic segmentation + dilation (1 pixel)	Automatic segmentation + dilation (2 pixel)
Femurs	DICE	0.882 ± 0.026	0.933 ± 0.012	0.974 ± 0.008	0.959 ± 0.013	0.920 ± 0.026
	Absolute mean of difference with manual FL (N)	473 ± 336	168 ± 105	119 ± 66	132 ± 121	203 ± 201
	Absolute mean of difference with manual FL (%)	7.42 ± 5.00	3.19 ± 2.55	2.25 ± 1.91	2.79 ± 3.37	4.45 ± 5.42
Vertebrae	DICE	0.763 ± 0.055	0.855 ± 0.050	0.929 ± 0.045	0.920 ± 0.043	0.862 ± 0.046
	Absolute mean of difference with manual FL (N)	2320 ± 449	832 ± 420	503 ± 505	500 ± 537	1126 ± 676
	Absolute mean of difference with manual FL (%)	38.69 ± 6.81	16.11 ± 9.11	7.53 ± 8.17	8.63 ± 11.18	19.00 ± 12.89

The results obtained when adding morphological operations are very similar to the previous ones. When adding a single erosion or dilation operation, the results are comparable in terms of failure load. When applying erosion operations, the resulting failure loads are lower than when using only the automated segmentation, whereas the failure load value tends to increase when adding dilations. Only the comparison between failure loads obtained with manual and automatic with 2 erosions showed statistical significance. Simulation results obtained for each femur and vertebra are detailed in Supplementary Tables S2 and S3.

The simulation results on vertebrae also show great similarity between the failure loads obtained with automatic and manual segmentations. The failure loads obtained with a single dilation operation are also comparable to those obtained with the manual segmentations. However, with added erosions or when adding a second dilation, the failure load drops and the difference with the ground-truth results is statistically significant in those three cases.

## Discussion

The objective of our approach was to quantify the effect of segmentation variation on failure load simulation. Our segmentation results are visually satisfactory for both femur and vertebrae segmentation, and the image metrics used show that our femur segmentation pipeline has results comparable to state-of-the-arts methods^23–25^, while being trained with a very small dataset. All areas of the femur including the hip joint are segmented with minimal error. Similarly, our vertebrae segmentation method produces results that closely match the performance of the top-performing techniques on the VerSe datasets^29^. Manual segmentations take at least 30 min to be done while the automatic approach only takes a few minutes, without the need of human intervention.

Our simulation results are in line with the anatomical reality, with the failure load being affected by the position of the metastasis, as well as the structure of the bone, with osteoporosis greatly influencing the simulations. The detailed results in Tables S2 and S3 in supplementary material show that the presence of metastasis tends to decrease substantially the failure load, but in some cases, due to the small size and favorable positioning of the metastasis, it does not impact the simulated failure load. This observation confirms the impact of the location and size of the metastasis on bone strength.

Our simulated failure loads show sensitivity to segmentation variations: When working on femurs, failure loads tend to increase slightly when applying dilation operations and decrease when applying erosions. It is usually obvious that adding material to the object increases strength and removing material decreases it. Indeed, the dilation operation will increase the volume of the area considered as our bone, and potentially add a few voxels corresponding to cortical bone that were not considered in the initial automatic segmentation. The erosion operations reduce the overall volume used in the simulation, and with less cortical bone considered, the failure load obtained decreases. The lower failure loads obtained after erosion were expected, but they were relevant to quantitatively assess the effect of a segmentation error on the simulation results. In the same way, the failure loads obtained after dilation are also very logical but provide additional information on the segmentation errors that can be deemed acceptable for a use in simulation. As seen in Table 3, while the DICE is slightly better with plain automatic segmentation, both the automatic segmentation and the segmentation with added dilation show simulation results really close to the ground-truth simulation. Results also show that an under-segmentation (missing some cortical pixels) impacts the results a lot more than an over-segmentation (more soft tissues are added which do not influence the failure load value). We could therefore consider post-processing methods accordingly, knowing that over-segmenting could help improve the simulation accuracy in some cases.

For vertebrae simulations, the resulting failure loads are a lot more sensitive to segmentation variations, but the results obtained with the plain automatic segmentation are very similar to those obtained using the manual segmentation. The obtained failure loads decrease as expected when adding erosion, but they decrease as well when adding dilation operations. This can be explained by the simulation method used for the simulations on vertebrae, with endplate detection being heavily reliant on the outside segmentation pixels. As seen in Table 3, the simulation results are closest to those obtained with the ground-truth when using plain automatic segmentation or automatic segmentation with a single dilation operation.

Failure load estimations could still be improved through optimization of the simulation protocol, and the next step would be to take into consideration specific mechanical properties of the metastasis. However, with only slight variations on the simulations for both vertebrae and femurs, we can assume that the precision attained with our segmentation models is sufficient to convert into accurate failure load simulations. The segmentations obtained with a single added dilation operation can also be of interest for failure load simulations and can provide additional information to the initial results on the considered bone.

## Materials and methods

In the following section, we detail the different datasets used for the two segmentation tasks and the simulations, as well as the simulation parameters and software used for failure load assessment. We also explain the pre-processing pipeline developed to be applied prior to the training phase of the neural networks. We describe our approach for simulation comparisons with manual and automatic segmentations.

The study protocol was approved by the French Ethics Committee (CPP SUD-EST 1 France) under registration number ID-RCB: 2019-A01202-55. All procedures have been conducted in compliance with national and European regulations. All included patients received clear information and provided written consent, and informed consent was obtained from all subjects and/or their legal guardian(s).

### Datasets

We use two datasets for bone segmentation: one publicly available for the vertebrae and one from the project for the femurs. Existing datasets with CT-scans of femur along with manual segmentation are either not available or lack the accuracy required for the training of robust models, especially on the femoral head. For the femur segmentation task, MEKANOS cohort was used (Hospices civils Lyon, agreement number N. 21 5467, May 28th, 2021, Supplementary Table S5). This cohort consists of eleven in-vivo CT-scans of hips, where both femurs are present. Those scans were acquired in clinical routine following a specific procedure (constant table height, quality phantom QA Mindways, 120 kV, 270 mAs, 1 Pitch, Field of view 360 mm and 200 mm, reconstruction: standard filter B, 512 × 512 matrix, slice thickness 0.7 mm) and with three manufacturers acquisition systems (General Electric, Philips and Siemens). A few femurs have metastatic osteolytic lesions which complicates the segmentation task but allows trained models to segment metastatic bones more efficiently, which is important for our study. From this database, eighteen femurs were manually segmented (4 femurs were not available).

As a secondary test dataset to better assess the robustness of the chosen trained model, additional femurs (n = 16, 9 patients) from four different centers were added from MEKANOS cohort a posteriori, along with manual segmentations. Inter-operator variability was also measured using 6 ex-vivo femurs (Supplementary Table S4). On those femurs, the impact of inter-operator variability on failure load is illustrated in Supplementary Fig. S2).

**Figure 3:**
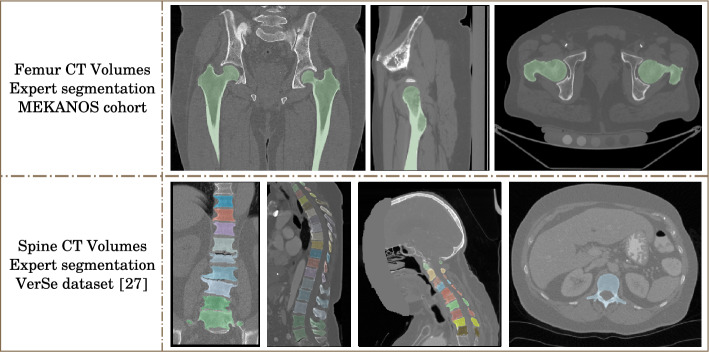
Example of CT data used for vertebrae and femurs segmentation.

For the vertebrae segmentation task, we used two publicly available datasets: VerSe2019 and VerSe2020^29^. Those datasets contain 374 CT-scans of various sizes all with manual segmentation. The number of vertebrae on each scan ranges from 3 to 25, with all types of vertebrae present in the dataset. In this study, we retained 363 patients, excluding those with additional transitional T13 scanned. Figure 3 shows examples of axial, coronal and sagittal slices taken from those datasets. The data used for simulation tests consists of 12 femurs from 6 patients (6 scans), among which 6 are healthy and 6 with metastasis, as well as 15 vertebrae from 2 patients (2 scans) among which 13 are healthy and 2 with metastasis (1 thoracic and 1 lumbar), all taken from the MEKANOS database.

### Simulation pipeline

**Figure 4:**
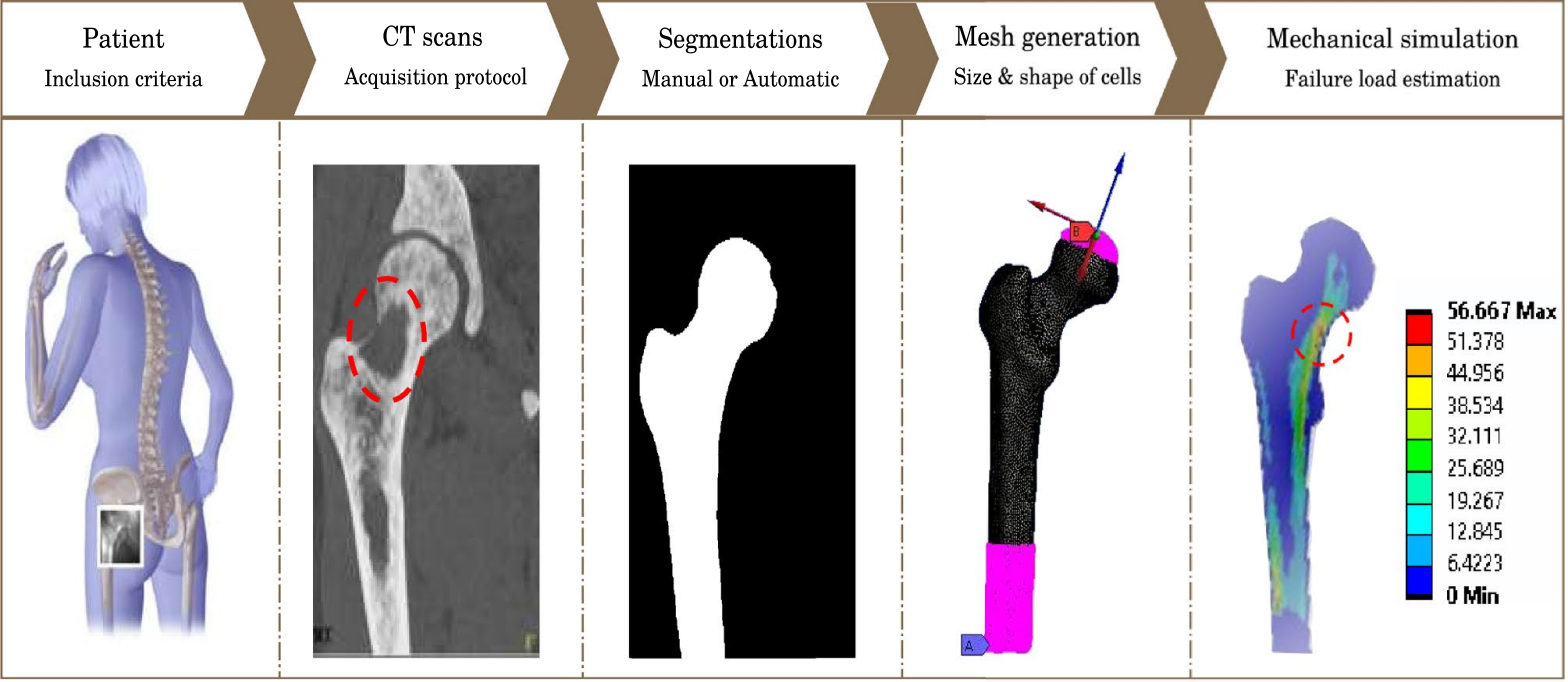
Simulation pipeline for fracture risk assessment.

For both vertebrae and femurs failure load simulations, a custom finite elements simulation pipeline, described in Fig. 4 was used with dedicated parameters in order to compute the failure load of the considered bones. When working on femurs, we used a published model from Sas et al*.* based on voxel-based hexahedral meshes^10^. These meshes were either obtained using a manual CT-segmentation using the software 3D Slicer, or using an automatic segmentation from a neural network described in part D. The intensity values in the CT-scan were converted to bone density using the calibration phantom included in the acquisition^10^. Each element of the mesh was attributed a bone density corresponding to the voxels bone densities. The bone density of each element was then used to compute the mechanical parameters of the non linear constitutive law^10^ (cf. Supplementary Fig. S1).

The grey density values were converted to Young’s modulus using the calibration phantom included in the acquisitions^10^:

1 $$E\left( {MPa} \right) = 14900\rho QCT^{1.86}$$

Using Ansys software (version 2021 R1), an axial compression was applied on the femurs, mimicking a standing position. An incremental displacement was applied on the top nodes of the femoral head (quasi-static) until it reached a maximal displacement. The nodes at the distal end of the diaphysis were constrained by a null displacement. The failure is defined as the maximum load occurring during the simulation^10^.

For vertebrae simulations, a quadratic tetrahedral mesh (10 nodes) with a volume of element of 1 mm^3^ was used based on experimental data from^33^, and for the numerical model, we used the elasticity-density relationship from^34^ using the calibration phantom:

2 $$E\left( {MPa} \right) = 3230\rho QCT - 34.7$$

We used a linear elastic—perfectly plastic constitutive law, with a yield strain of 1.5% strain^35^. The failure criteria consisted in considering a strain of 1.9% of the total vertebral height reduction^36^. All the simulations were also run using Ansys software.

### Pre-processing pipeline

**Figure 5:**
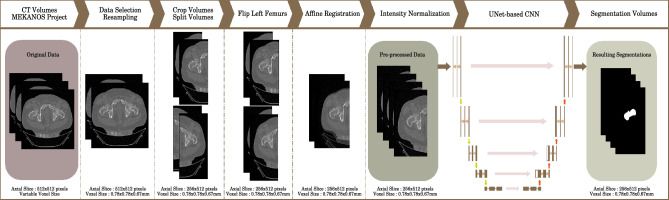
Segmentation pipeline used for femur segmentation.

For femur segmentation, we propose a fully automated segmentation method with a pre-processing pipeline as illustrated in Fig. 5 to facilitate the deep learning training. Our dataset contains only few annotated data, and dedicated pre-processing is important to ensure the robustness of the proposed models.

The pre-processing pipeline is made of several steps: after selection and manual expert annotation of the femurs, the volumes are all resampled to the median voxel size (0.78 × 0.78 × 0.67 mm), then cropped when both femurs are present in order to separate them into two distinct volumes. The split left femurs are then flipped to obtain a comparable orientation for all volumes. To further increase the homogeneity of the dataset, especially the spatial orientation of the femurs, the flipped left femurs and right femurs are registered together, using affine transforms. Adding the co-registration step between femurs makes the global spatial orientation more similar between femurs. Co-registration ensures the robustness of the network despite few training data, while preserving the automatic aspect of our pipeline. The resulting volumes are then all normalized before being used as input of the convolutional neural network.

In addition to ensuring the proper training of the neural network, the pre-processing pipeline allows to increase the size of the dataset, thanks to the splitting of the initial volumes.

### Neural networks

We used several convolutional neural networks all based on the U-Net architecture^31^. We implemented a 2D multi-planar U-Net, as well as a 3D U-Net for femur segmentation. We compared the results with nnUNet, the state-of-the-art convolutional neural network for medical image segmentation^32^.

Three 2D-UNet were trained on axial, coronal and sagittal slices for 500 epochs. The 3 resulting segmentations were then fused using majority voting. The 3D U-Net model was trained using random patches of size 64 × 64 × 64 for 300 epochs. Data augmentation, such as random rotations, translations, shearing and scaling was used on-the-fly to prevent overfitting. All custom UNets were trained using Adam optimizer (β_1_ = 0.9, β_2_ = 0.999) and a DICE loss, with a learning rate α = 2 × 10^−4^ and a batch size of 16 for 2D U-Net, α = 3 × 10^−5^ and a batch size of 4 for 3D U-Net. We also added morphological post-processing operations based on binary dilation and erosion to remove small unwanted islands and improve segmentation results.

The nnUNet architecture used is the ‘3d fullres’, with patch sizes automatically selected (238 × 196 × 208 for femur segmentation and 205 × 205 × 205 for vertebrae segmentation) and default parameters, and was trained for 1000 epochs. The optimizer used is stochastic gradient descent with an initial learning rate of 0.01. The batch size was set to 2 for both trainings. We only used this architecture for vertebrae segmentation as the results for femur segmentation were comparable to our custom 3D U-Net and the amount of training data was sufficient to avoid the need for dedicated pre-processing, and the only pre-treatments operations were automatically made with nnUNet.

The models were trained on a Nvidia P100 GPU with 16 GB VRAM. The total training time was 12 h for 2D U-Net per axis, 16 h for 3D U-Net and 48 h for nnUNet on the femur dataset. This substantial difference is also present during inference, where nnUNet takes up to 30 min for a prediction when standard models only take up to 3 min.

### Segmentations and simulations comparison

To quantify the segmentation results, we used the Sørensen-Dice score (noted DSC) to evaluate the similarity between the ground-truth and the automatic segmentations ([0;1] where 1 is the best), as well as the Hausdorff distance (noted HD) to evaluate the maximum errors of the automatic segmentations (in mm, smaller the better). All metrics are computed on 3D volumes. We used a fivefold cross-validation to quantify more accurately the results. Among the 18 available femurs, 12 were used for training, 4 for validation and 2 for testing. For vertebrae segmentation, 242 scans were used for training, 61 for validation and 60 for testing.

To compare the influence of the segmentation on the failure load simulations, we computed the failure load on 12 femurs, using automatic segmentations and using expert manual annotation for comparison. We also compared results using automatic and manual segmentation on 15 thoracic and lumbar vertebrae. In both cases, we also applied simple morphological operations (dilation/erosion), with either one or two iterations to the automatic segmentation as a way to introduce variability to the automatic segmentations. The objective is to investigate the effect of slight segmentation variations on the resulting failure load.

### Statistical analysis

Statistical tests were performed using SPSS software (SAS Institute, Cary, NC). Differences among groups were evaluated using non-parametric test (Friedman test). When a significant overall F value (*P* < 0.05) was present, differences between individual group means were tested using Dunn’s multiple comparison post-hoc tests. Only comparisons with the manual segmentation are presented. For all tests, *P* < 0.05 was considered statistically significant. Data are presented as mean ± standard error.

## Conclusion

In this paper we proposed a dedicated pre-processing pipeline for femur segmentation as well as deep learning based segmentation methods for femurs and vertebrae segmentation. From our experiments, we showed that it is primordial to use pre-processing in order to improve the segmentation results. U-Net architectures are efficient and can serve as primary tools to perform automated bone segmentations. We showed that failure load simulations depend on the initial segmentations, and that automatic segmentations yield similar simulation results as simulations made with expert segmentations. The variations on segmentations when adding dilation or erosion operations impact the simulations, with the closest results to the simulations using expert simulations being those using the plain automatic segmentation and the automatic segmentation with one dilation operation. The results obtained allow us to envision the use of this approach in a broader pipeline in biomechanical simulations on patients with metastatic lesions.

### Supplementary Information

Supplementary Information.

## Data Availability

Data is not made publicly available due to ethical or privacy reasons. Data used for this study is available on request to the corresponding author.
