# Supplementary material for: Finite element models with automatic computed tomography bone segmentation for failure load computation
Source: Sci Rep. 2024 Jul 17;14:16576. doi: 10.1038/s41598-024-66934-w (PMC11255209; doi:10.1038/s41598-024-66934-w)

**Supplementary material**

SUPPLEMENTARY TABLE S1: Metrics obtained for each vertebra in the test set

| Vertebra — Number of cases | DC | HD (mm) |
| --- | --- | --- |
| C1 — 14 | 0.85±0.06 | 9.50±5.33 |
| C2 — 14 | 0.95±0.01 | 3.19±1.10 |
| C3 — 14 | 0.94±0.01 | 2.63±0.65 |
| C4 — 15 | 0.88±0.21 | 3.58±3.84 |
| C5 — 16 | 0.88±0.14 | 5.86±4.65 |
| C6 — 18 | 0.91±0.05 | 6.74±5.46 |
| C7 — 24 | 0.92±0.05 | 7.02±6.29 |
| T1 — 31 | 0.94±0.02 | 5.84±6.05 |
| T2 — 32 | 0.94±0.03 | 5.22±4.93 |
| T3 — 28 | 0.93±0.04 | 5.95±5.28 |
| T4 — 25 | 0.90±0.16 | 7.91±7.93 |
| T5 — 25 | 0.86±0.23 | 9.96±9.09 |
| T6 — 24 | 0.82±0.29 | 10.92±10.15 |
| T7 — 26 | 0.78±0.33 | 12.58±12.49 |
| T8 — 28 | 0.72±0.40 | 12.61±11.19 |
| T9 — 32 | 0.76±0.37 | 10.31±10.59 |
| T10 — 39 | 0.81±0.33 | 9.76±9.99 |
| T11 — 40 | 0.85±0.28 | 8.78±10.49 |
| T12 — 37 | 0.91±0.21 | 7.36±9.21 |
| L1 — 42 | 0.91±0.20 | 9.46±11.53 |
| L2 — 43 | 0.90±0.17 | 9.84±12.60 |
| L3 — 43 | 0.88±0.20 | 12.56±14.25 |
| L4 — 43 | 0.85±0.26 | 20.08±41.66 |
| L5 — 42 | 0.82±0.31 | 26.34±59.34 |
| L6 — 5 | 0.78±0.40 | 16.74±23.71 |

SUPPLEMENTARY TABLE S2: Simulated femoral failure load depending on segmentation

|  | | Failure Load (N) | | | | | |
| --- | --- | --- | --- | --- | --- | --- | --- |
| Patient | Side — Metastasis | Manual | Automatic  + erosion (n=2) | Automatic  + erosion (n=1) | Automatic segmentation | Automatic  + dilation (n=1) | Automatic  + dilation (n=2) |
| 1 | Left — No | *8077* | 6832 | 7889 | 7989 | **8045** | 8676 |
|  | Right — Yes | *5881* | 4943 | 5510 | **6029** | 6052 | 6081 |
| 2 | Left — No | *2185* | **2171** | 2357 | 2438 | 2444 | 2603 |
|  | Right — Yes | *2001* | 1748 | 1864 | 1956 | **2040** | 2076 |
| 3 | Left — Yes | *8322* | 7826 | 8016 | 8169 | 8083 | **8223** |
|  | Right — No | *8426* | 8159 | **8411** | 8478 | 8509 | 8607 |
| 4 | Left — Yes | *6403* | 5848 | **6212** | 6662 | 6815 | 6939 |
|  | Right — Yes | *5694* | 5258 | 5629 | 5621 | **5687** | 5766 |
| 5 | Left — No | *9739* | 9262 | 9674 | 9716 | **9761** | 9654 |
|  | Right — Yes | *9708* | 9505 | **9827** | 9863 | 9860 | 9833 |
| 6 | Left — Yes | *5951* | 5681 | 5838 | 5841 | 6030 | **5946** |
|  | Right — No | *7311* | 6785 | 7043 | 7156 | 7226 | **7349** |
| Absolute difference from simulation with manual segmentation (%) | |  | 7.4 | 3.2 | **2.3** | 2.8 | 4.4 |

SUPPLEMENTARY TABLE S3: Simulated vertebral failure load depending on segmentation

|  | | Failure Load (N) | | | | | |
| --- | --- | --- | --- | --- | --- | --- | --- |
| Patient | Vertebra— Metastasis | Manual | Automatic  + erosion (n=2) | Automatic  + erosion (n=1) | Automatic segmentation | Automatic  + dilation (n=1) | Automatic  + dilation (n=2) |
| 1 | T8 — No | *4187* | 2327 | 3256 | 4145 | **4228** | 3877 |
|  | T9 — No | *4846* | 2942 | 4170 | 5206 | 5219 | **4624** |
|  | T10 — No | *5480* | 3247 | 4614 | 5614 | **5493** | 4865 |
|  | T11 — Yes | *7539* | 4861 | 6843 | 8206 | **7988** | 6917 |
|  | T12 — No | *7918* | 4791 | 6469 | **7834** | 7821 | 6952 |
|  | L1 — No | *5198* | 2510 | 3809 | 4869 | **4909** | 4222 |
|  | L2 — No | *5361* | 2895 | 4282 | **5254** | 3556 | 2312 |
|  | L3 — No | *5311* | 3188 | 4379 | **5435** | 5141 | 4179 |
|  | L4 — No | *3772* | 5087 | 2475 | **4912** | 5131 | 5015 |
|  | L5 — No | *5538* | 3560 | 4798 | 5768 | **5605** | 4631 |
| 2 | L1 — No | *6776* | 3962 | 5640 | **6839** | 6427 | 4970 |
|  | L2 — No | *7254* | 4728 | 6651 | 7825 | **7360** | 5880 |
|  | L3 — No | *7560* | 5158 | 6983 | 8382 | **8011** | 6482 |
|  | L4 — No | *8548* | 6343 | **8647** | 10249 | 9618 | 7162 |
|  | L5 — Yes | *8998* | 6516 | **8990** | 10176 | 9855 | 7797 |
| Absolute difference from simulation with manual segmentation (%) | | | 38.7 | 16.1 | **7.5** | 8.6 | 19.0 |

SUPPLEMENTARY TABLE S4: Expert segmentation variability and comparison with automatic segmentation

|  | **Mean DICE score** |
| --- | --- |
| Expert vs Expert | 0.973 ± 0.013 |
| Expert vs Automatic | 0.980 ± 0.008 |

The DICE obtained between experts is comparable to the DICE between expert segmentation and automatic segmentation. Manual segmentation of femurs takes at least 30 minutes using semi-automatic tools such as region growing, thresholding and interpolation. Comparatively, the automatic approach takes only a few minutes, without the need for human intervention.

SUPPLEMENTARY TABLE S5: Descriptive statistics on patients

|  | **PATIENT Number** | **GENDER** | **AGE at scan (yrs)** | **Height (cm)** | **Weight (kg)** | **BMI** |
| --- | --- | --- | --- | --- | --- | --- |
| **Initial Set-up** | 1-003 | Male | 74 | 155 | 46 | 19,1 |
|  | 1-005 | Male | 68 | 185 | 108 | 31,6 |
|  | 1-006 | Male | 61 | 167 | 67 | 24,0 |
|  | 1-007 | Female | 41 | 165 | 64 | 23,5 |
|  | 1-008 | Male | 72 | 169 | 70 | 24,5 |
|  | 1-011 | Male | 69 | 180 | 77 | 23,8 |
|  | 1-012 | Female | 64 | 160 | 43 | 16,8 |
|  | 1-014 | Female | 52 | 152 | 57 | 24,7 |
|  | 1-015 | Male | 61 | 176 | 70 | 22,6 |
|  | 1-019 | Female | 83 | 160 | 56 | 21,9 |
|  | 1-023 | Male | 76 | 174 | 85 | 28,1 |
|  | **Mean** |  | **66** | **168** | **68** | **23,7** |
|  | **SD** |  | **12** | **10** | **18** | **3,9** |
| **2nd Set-up** | 08-001 | Male | 81 | 171 | 77 | 26,3 |
|  | 09-001 | Male | 73 | **170** | **68** | 23,5 |
|  | 12-002 | Female | 61 | 155 | 52 | 21,6 |
|  | 12-006 | Female | 65 | 147 | 54 | 25,0 |
|  | 1-029 | Female | 77 | 155 | 52 | 21,6 |
|  | 1-030 | Male | 76 | 181 | 75 | 22,9 |
|  | 1-034 | Male | 57 | 173 | 63 | 21,0 |
|  | 1-043 | Male | 75 | 169 | 69 | 24,2 |
|  | 1-047 | Male | 79 | 168 | 64 | 22,7 |
|  | **Mean** |  | **72** | **165** | **64** | **23,2** |
|  | **SD** |  | **8** | **11** | **9** | **1,7** |
| BMI: Body Mass Index | |  |  |  |  |  |
| Initial set-up served for training, validation and 1st validation test | | | | | |  |
| 2nd set-up served only for test | | |  |  |  |  |

SUPPLEMENTARY FIGURE S1: A/ Non linear constitutive law of the model. B/ Each element has specific values of the parameters depending on their bone density. (Adapted from Keyak et al. 2005)

*J. H. Keyak, T. S. Kaneko, J. Tehranzadeh, et H. B. Skinner, « Predicting proximal femoral strength using structural engineering models », Clin Orthop Relat Res, nᵒ 437, p. 219‑228, august 2005, doi: 10.1097/01.blo.0000164400.37905.22.*

| 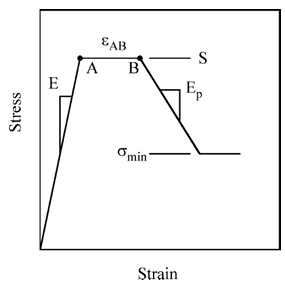 | 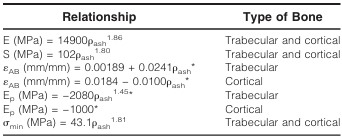 |
| --- | --- |
| A/ | B/ |

SUPPLEMENTARY FIGURE S2: Simulated failure loads obtained on 6 ex-vivo femurs using different segmentations from 4 operators (beginner or expert) or automatic segmentation


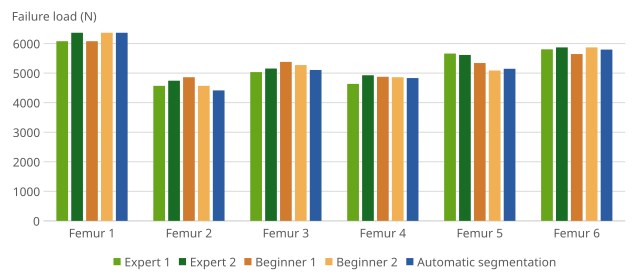

Supplement: Supplementary file 1 — Supplementary Information. [file 41598_2024_66934_MOESM1_ESM.docx]
